# Could an Impairment in Local Translation of mRNAs in Glia be Contributing to Pathogenesis in ALS?

**DOI:** 10.3389/fnmol.2019.00124

**Published:** 2019-05-21

**Authors:** Samantha K. Barton, Jenna M. Gregory, Siddharthan Chandran, Bradley J. Turner

**Affiliations:** ^1^Florey Institute of Neuroscience and Mental Health, The University of Melbourne, Parkville, VIC, Australia; ^2^Euan MacDonald Centre for MND Research, University of Edinburgh, Edinburgh, United Kingdom; ^3^Centre for Clinical Brain Sciences, University of Edinburgh, Edinburgh, United Kingdom; ^4^UK Dementia Research Institute at University of Edinburgh, Edinburgh, United Kingdom

**Keywords:** RNA trafficking, local translation, ALS, oligodendrocytes, astrocytes

## Abstract

One of the key pathways implicated in amyotrophic lateral sclerosis (ALS) pathogenesis is abnormal RNA processing. Studies to date have focussed on defects in RNA stability, splicing, and translation, but this review article will focus on the largely overlooked RNA processing mechanism of RNA trafficking, with particular emphasis on the importance of glia. In the central nervous system (CNS), oligodendrocytes can extend processes to myelinate and metabolically support up to 50 axons and astrocytes can extend processes to cover up to 100,000 synapses, all with differing local functional requirements. Furthermore, many of the proteins required in these processes are large, aggregation-prone proteins which would be difficult to transport in their fully translated, terminally-folded state. This, therefore, highlights a critical requirement in these cells for local control of protein translation, which is achieved through specific trafficking of mRNAs to each process and local translation therein. Given that a large number of RNA-binding proteins have been implicated in ALS, and RNA-binding proteins are essential for trafficking mRNAs from the nucleus to glial processes for local translation, RNA misprocessing in glial cells is a likely source of cellular dysfunction in ALS. To date, neurons have been the focus of ALS research, but an intrinsic deficit in glia, namely astrocytes and oligodendrocytes, could have an additive effect on declining neuronal function in ALS. This review article aims to highlight the key evidence that supports the contention that RNA trafficking deficits in astrocytes and oligodendrocytes may contribute to in ALS.

## Introduction

Amyotrophic lateral sclerosis (ALS) and frontotemporal dementia (FTD) are characterized pathologically by the accumulation of cytoplasmic inclusions in affected neurons and glial cells that, in 97%–98% of cases, stain positive for TAR-DNA binding protein 43 (TDP-43; Neumann et al., 2006). TDP-43, like many of the proteins that have been associated with ALS pathogenesis, is a DNA and RNA-binding protein, implicating impaired RNA processing as a possible mechanism of disease.

Regulatory proteins bind to mRNA transcripts to aid in localization, translation and stability, whilst also playing important roles in transcription, alternative splicing and nuclear export. The binding of regulatory proteins to mRNAs, as well as the accrual of various translation factors, forms an RNA granule. Dysregulation of RNA granule formation has been implicated in neurodegenerative disease (Buchan, 2014), including ALS and FTD (Fan and Leung, 2016). Their involvement ranges from an impaired ability of the RNA granule to sufficiently aid in localization, translation and stability of the mRNAs, to the formation of stress granules, all of which have been implicated in ALS and can initiate a cascade of downstream events predicted to play a role in neuronal decline (Fan and Leung, 2016). This review article will focus specifically on impaired mRNA localization, which is due to compromised nucleocytoplasmic export and impaired trafficking to glial processes.

Subcellular transportation of mRNAs provides cells with the ability to translate proteins in response to localized signals. Local translation has been extensively documented in neuronal cell types and is noted to be essential for their normal function as it allows a high level of cellular autonomy from the cell soma to the dendrites, particularly during axonal pathfinding (Yoon et al., 2009). It is also established that glia within the central nervous system (CNS) undergo local translation. The cellular complexity of glia coupled with their elaborate roles in the CNS (ranging from input into vascular control to providing physical, trophic and metabolic support to neurons to the stabilization of synapses) renders the requirement for local translation essential to allow the translation of proteins unique to the specific function of each glial process. Further, the dynamic nature of processes like myelination and synaptic activity means that cells can respond more readily. These concepts have not been extensively explored in disease models. The aggregation of RNA-binding proteins, and associated proteins involved in RNA trafficking, in pathological inclusions in ALS is well established and so this review article aims to detail what impact these aggregations could have on local translation of key mRNAs in glia. We propose that the proteinopathy present in the majority of ALS patients could be impacting on local translation of mRNAs, thereby hindering the normal function of glia in patients with ALS and FTD, potentially contributing to neuronal decline and disease progression.

## Glial Involvement in ALS

**Figure 1:**
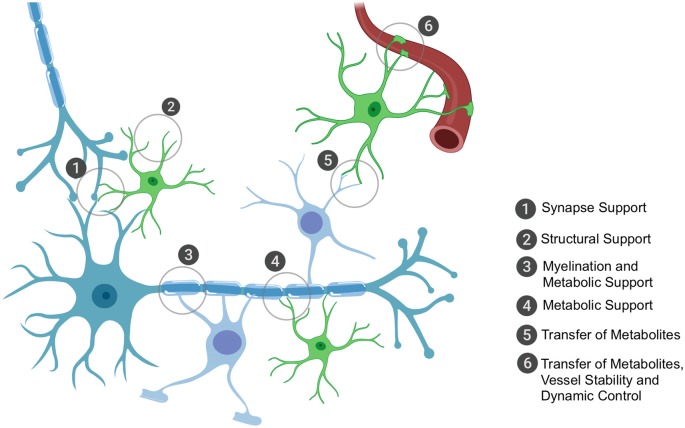
The key roles of oligodendrocytes and astrocytes in the brain that rely on local translation of mRNAs. Astrocytes and oligodendrocytes have the ultimate role of supporting neuronal function. Astrocytes have a plethora of functions that range from structurally supporting synapse formation (1) to providing general structural support in the central nervous system (CNS) (2), especially in development during axon formation and pathfinding. Oligodendrocytes have the metabolically demanding function of myelinating axons for accelerated conductance (3) and it is through these myelin sheaths that oligodendrocytes can shuttle metabolic and trophic factors to neurons (3). Astrocytes are also critical in providing metabolic and trophic support to neurons (4) and can also shuttle metabolites to oligodendrocytes for their own use or to shuttle onwards to neurons (5). Astrocytes are also important for blood vessel stability and assist in tight junction formation; they also can receive glucose from the bloodstream (6) and can shuttle this to oligodendrocytes or themselves convert it to lactate (the end-product of glycolysis of glucose and the energy substrate for neurons) and shuttle this to oligodendrocytes as well or directly to neurons. Importantly, the key sites of support to neurons occur at the distal processes, thus local translation of mRNAs is critically important to ensure these processes are dynamic and in rapid response to the surrounding environment’s requirements.

Humans have the highest proportion of glial cells, with their primary role being to support neuronal function, whether that be *via* structural support, metabolic and trophic support or through protection from surrounding threats to neuronal health. Broadly, glial cells encompass oligodendrocytes, astrocytes and specialized immune cells called microglia. For the purpose of this review article, we will focus on non-immune glial cells; their key roles (all of which depend on effective mRNA trafficking and local translation) are highlighted in Figure 1.

Given the importance of glia as neuronal support cells, intrinsic glial dysfunction has been implicated in neurodegenerative diseases including ALS (Yamanaka et al., 2008; Kang et al., 2013; Serio et al., 2013; Ferraiuolo et al., 2016; Madill et al., 2017). In the TDP-43^Q331K^ mouse model, it was shown that selective removal of the mutation from motor neurons alone did not prevent motor neuron or neuromuscular junction loss and did not reduce the activation of microglia or astrocytes (Ditsworth et al., 2017). More specifically, it has been shown that co-culturing induced pluripotent stem cell (iPSC)-derived oligodendrocytes from sporadic ALS patients, as well as those harboring mutations in *C9orf72* and *TDP-43*, with HB9-GFP motor neurons led to significant motor neuron loss compared to co-cultures with iPSC-derived oligodendrocytes from healthy control patients (Ferraiuolo et al., 2016). Similarly, a detrimental effect of co-cultured astrocytes (derived from neural precursor cells isolated from post-mortem tissue of sporadic ALS patients) on mouse embryonic stem cell-derived motor neurons survival was also observed (Haidet-Phillips et al., 2011). Thus, glia are critically involved in ALS pathogenesis.

The majority of research into glial influence in ALS and FTD to date has been on what impact glial dysfunction has on neuronal health. This is obviously relevant given that the symptoms experienced by ALS patients are related to the loss of upper and lower motor neurons. However, it is important to also address the intrinsic glial deficit. What tends to be underappreciated is that glia have the metabolically demanding role of not only sustaining their own functions but are also required to support the surrounding environment. Astrocytes and oligodendrocytes, in particular, are required to metabolically support neuronal function by directly shuttling lactate to neurons as their primary fuel source (Dienel and Cruz, 2004; Fünfschilling et al., 2012; Saab et al., 2013, 2016). Thus, any process that amplifies metabolic demand, or interferes with normal metabolic processes, would have a detrimental effect directly on cellular function and indirectly on surrounding neurons. So, we suggest that impaired RNA processing in glia could be contributing to ALS and FTD pathogenesis.

## ALS-Linked RNA-Binding Proteins

Many known genetic mutations linked with ALS and FTD are in genes involved in maintaining RNA homeostasis. Examples include *TARDBP* (encoding TDP-43), FUS RNA-binding protein (*FUS*), *C9orf72* and, to a lesser extent, heterogeneous nuclear ribonucleoproteins A1 (*hnRNP A1*) and A2/B1 (*hnRNP A2/B1*), EWS RNA-binding protein 1 (*EWSR1*), angiogenin (*ANG*), senataxin (*SETX*), matrin 3 (*MATR3*) ataxin-2 (*ATXN2*) and TATA-box binding protein associated factor 15 (*TAF15*; Ling et al., 2013; Kapeli et al., 2017). Many of these proteins have both loss-of-function and gain-of-function toxic effects on cells. For example, iPSC-derived motor neurons from patients harboring a mutation in the *hnRNP A2/B1* gene not only lost the function of this protein but also gained toxicity linked to failure in RNA processing (Martinez et al., 2016). The hypothesis that RNA-binding proteins aggregating in the cytoplasm of neurons being a pathogenic mechanism in ALS is not a new concept and has been suggested, and reviewed (Yasuda and Mili, 2016), previously. Briefly, it is suggested that the aggregation of RNA-binding proteins limits RNA transport along neuronal microtubules thus preventing local translation at the dendrites and even leading to ectopic translation in the cell body. RNA trafficking in glia in ALS remains relatively unexplored but we predict similar processes could apply and contribute to cellular pathogenesis. The current understanding of these processes is detailed below.

**Figure 2:**
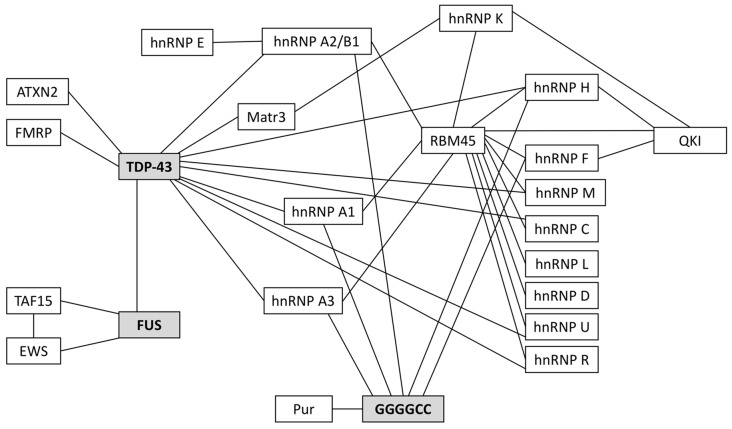
Protein binding partners that have been implicated in ALS. 97–98% of ALS patients have pathogenic protein inclusions that stain positive for the RNA/DNA binding protein TDP-43. Most of the remaining 2%–3% of patients will have inclusions that stain positive for FUS. Currently, the most prevalent genetic mutation associated with familial and sporadic ALS is the *C9orf72* hexanucleotide repeat expansion (HRE); these patients characteristically have TDP-43-positive inclusions and/or TDP-43-negative, ubiquitin-positive inclusions and/or pathogenic RNA foci (composed of GGGGCC repeats), all of which are known to sequester RNAs and proteins. Many of the RNA-binding proteins in this schematic are critical for RNA trafficking and local translation of key mRNAs in astrocytes and oligodendrocytes.

Many of the ALS-associated pathogenic proteins are known to be essential for nucleo-cytoplasmic mRNA trafficking and, importantly, many will interact with one another for efficient RNA metabolism. These interactions are highlighted in Figure 2; they have been reviewed elsewhere (Dormann and Haass, 2013; Vanderweyde et al., 2013; Balendra and Isaacs, 2018) so we will detail only those of relevance to glial mRNA trafficking.

TDP-43 has multiple roles in RNA processing and is known to bind to more than 6,000 RNA targets, equating to approximately 30% of the human transcriptome (Ling et al., 2013); it is topologically and functionally dysregulated in 97% of ALS patients (Neumann et al., 2006, 2007; Seilhean et al., 2009; Murray et al., 2011; Armstrong, 2017). FUS binds to over 5,000 RNA targets in the brain (Ling et al., 2013) but is topologically and functionally dysregulated in <1% of ALS patients and 9% of FTD patients (Blokhuis et al., 2013; Ling et al., 2013). Both TDP-43 and FUS have been associated with hnRNP A1 (Kamelgarn et al., 2016) with TDP-43 specifically linked with aberrant splicing of *hnRNP A1* (Deshaies et al., 2018) and altered expression and aberrant splicing of *hnRNP A2/B1* (Highley et al., 2014). Coady and Manley (2015) used the human glioblastoma cell line, U87, to show that mutations in *FUS* can lead to reduced MeCP2 levels (a transcription factor regulator); reduced MeCP2 protein levels in glia has previously been shown to be toxic to neurons leading to altered dendritic morphology and, ultimately, neuronal death (Ballas et al., 2009).

The most common genetic mutation underlying ALS and FTD is the *C9orf72* hexanucleotide repeat expansion (HRE), which pathologically presents as G_4_C_2_ repeat RNA foci and sense and anti-sense repeat-associated non-ATG (RAN) translation of di-peptide repeat proteins (DPRs) as well as TDP-43 aggregation. G_4_C_2_ repeat RNA foci co-localize with various RNA-binding proteins in neurons, including hnRNP A1 and hnRNP H/F, shown in post-mortem tissue (Cooper-Knock et al., 2014) and in cell lines, primary neurons and zebrafish embryos (Lee et al., 2013; Conlon et al., 2016). Of relevance, this has also been validated in human astrocytes derived from the autopsied motor cortex (Conlon et al., 2016). In addition to being an RNA-binding protein, hnRNP H is a key splicing regulator; notably, hnRNP H splices transcripts encoding other splicing proteins, which includes TDP-43 and FUS (Uren et al., 2016). Other RNA and nucleic acid-binding proteins known to bind to the G_4_C_2_ repeats are hnRNP A3, which is important for nuclear export of mRNAs (Mori et al., 2013). DPRs have been more challenging to characterize given the limited access to efficient antibodies. *In vitro* work has demonstrated that DPRs are highly prone to aggregation and can sequester proteins, including RNA-binding proteins, which could contribute to abnormal RNA trafficking in neurons and glia (Gendron et al., 2013; Porta et al., 2015). DPRs can dock at the nuclear pore and disrupt the architecture of the nuclear envelope leading to limited nuclear import and export of macromolecules and mRNAs (Jovičić et al., 2015; Zhang et al., 2016; Shi et al., 2017). Both poly(GR) and poly(PR), two of the five DPRs, have been shown to interact with TDP-43-positive RNA as well as stress granules and are predicted to be involved in the fibrillization of TDP-43 and other RNA-binding proteins such as hnRNP A2/B1 (Molliex et al., 2015; Freibaum and Taylor, 2017). Importantly, Edbauer and colleagues demonstrated that poly(GA) can cause mislocalization of TDP-43 in neurons (Khosravi et al., 2017).

## RNA Trafficking in Astrocytes

Astrocytes are the most abundant cell type in the human brain, with functions including: (i) neuronal support, including metabolic, trophic and structural support; (ii) neuronal synapse and axon formation; and (iii) regulation of cerebral blood flow (Blackburn et al., 2009). Their role in neurodegeneration remains contentious with astrocytes believed to have both protective and harmful effects (Phatnani and Maniatis, 2015), much like microglia (Geloso et al., 2017). Astrocytes have a complex morphology; in cerebral gray matter their processes can span 50 μm and up to 300 μm in the white matter, rendering local translation necessary for normal cellular function. There is also a high metabolic demand on astrocytes, both for their own function as well as metabolically supporting neurons; one astrocyte can be responsible for being in contact with up to 100,000 synapses (Sakers et al., 2017) covering a surface area of between 60,000 and 80,000 μm^2^. The dynamic nature of astrocytic function means these cells have optimized their protein availability relying heavily on local translation (Boulay et al., 2017).

Glial fibrillary acidic protein (*GFAP*) mRNA is known to be translated locally at astrocytic processes and is an abundantly expressed protein in all astrocytes, despite the known heterogeneity among the astrocyte population. To further validate this, it has been shown to equate to 15% of an astrocyte’s volume and the protein expression is isolated predominantly to its processes (Bushong et al., 2002). GFAP-null mice ranged in phenotype, but half of the cohort were seen to have extensive deficits including abnormal myelination, reduced white matter vascularization and decreased integrity of the blood brain barrier (Liedtke et al., 1996). The RNA-binding protein responsible for shuttling *GFAP* to the peripheral astrocyte processes (PAP) is Quaking (QKI; Sakers et al., 2017). QKI is abundantly expressed in oligodendrocytes and astrocytes and has been implicated in schizophrenia (Aberg et al., 2006) and brain cancers (Molenaar et al., 2012) but is yet to be associated with ALS pathology. However, given it is known to bind to 2,500 mRNAs, as well as its close relationship to other affected RNA-binding proteins (it contains a hnRNP K homology domain, an hnRNP that has been implicated in ALS; Hafner et al., 2010), it deserves further investigation. Another key astrocytic mRNA known to be locally translated is excitatory amino acid transporter-2 (*EAAT2*) and whilst its trafficking has not been explored, *EAAT2* transcripts with both intron-retention and exon-skipping have been found in 65% of sporadic ALS patients in neuropathologically affected areas as well as in the cerebrospinal fluid (CSF; Lin et al., 1998). Other mRNAs known to be translated locally include *Aqp4* (encoding the main water channel protein in the CNS responsible for maintaining water homeostasis), *Aldh1a1* (encoding an enzyme that depicts mature astrocytes; Boulay et al., 2017), *Slc1a2*, *Slc1a3* and *GluI* (encoding enzymes important for glutamate metabolism), *Fads1*, *Fads2*, *Scd1, Scd2, Elovl5* and *Hadha* (encoding enzymes critical for fatty acid synthesis), *Kif1c* and *Myo1D* (encoding proteins that maintain the cytoskeleton), *Mertk, Sparc* and *Thbs4* (encoding proteins key for synapse regulation), *ApoE* (encoding a class of proteins required for fat metabolism) and *CLU* (encoding the chaperone protein clusterin that has been implicated in ALS; Sakers et al., 2017).

Thus, if these mRNAs failed to adequately translate, this would have a detrimental impact on neuronal function given that they are critically involved in supportive pathways like metabolism and synapse regulation. Further, not only could mRNAs become sequestered *via* their RNA-binding proteins, but key astrocytic proteins are also sequestered, impeding function. TDP-43 is a binding partner for EAAT2, the glutamate transporter introduced above, and it has been shown that TDP-43 aggregation correlates with decreased EAAT1/2 glutamate transporter levels in the spinal cords of both an ALS mouse model (Tong et al., 2013) and human patients (Rothstein et al., 1995). FMRP is a known binding partner of TDP-43; in patients with Fragile × syndrome (whereby they have a repeat-expansion in the *FMR1* gene and acquire FMRP inclusions), profiling these inclusions demonstrated that many astrocytic proteins have been associated with these FMR inclusions, including vimentin, GFAP, and hnRNP A2/B1 [and, interestingly, myelin basic protein (MBP; Iwahashi et al., 2005)] Given FMRP is a known binding partner of TDP-43, these protein associations are relevant to ALS.

Thus, the sequestration of key RNA-binding proteins in astrocytes is likely to have a detrimental effect by impeding the trafficking of mRNAs critical for cellular function to their site of local translation.

## RNA Trafficking in Oligodendrocytes

Oligodendrocytes have been underappreciated for their role in the CNS with the focus to date limited to their role in myelination. Recent research has uncovered their ability to metabolically support neurons through the shuttling of lactate (Fünfschilling et al., 2012; Saab et al., 2013, 2016), much like astrocytes, highlighting a critical role for oligodendrocytes in neuronal health, function and survival. Each oligodendrocyte has the capacity to produce up to 50 myelin sheaths, each of which can be up to 60 μm in length (Hughes et al., 2018), so their support and metabolic supply (and demand) is extensive.

It has long been established that many mRNAs in oligodendrocytes are translated locally at the myelin sheath (Campagnoni et al., 1980; Colman et al., 1982) including *MBP* (Kristensson et al., 1986; White et al., 2008), myelin-associated oligodendrocytic basic protein (*MOPB*; Holz et al., 1996), carbonic anhydrase II (*CAII*; Ghandour and Skoff, 1991), *tau* (LoPresti et al., 1995) and amyloid precursor protein (*APP*; Garcia-Ladona et al., 1997). This extends to the involvement of many RNA-binding proteins including hnRNP A2/B1, hnRNP A1, hnRNP E1, hnRNP H/F and hnRNP K, to name a few (White et al., 2008; Laursen et al., 2011; Torvund-Jensen et al., 2014).

hnRNP A1 and hnRNP A2/B1 are involved in *MBP* mRNA trafficking and both are also known binding partners of G_4_C_2_ repeats and TDP-43 (Highley et al., 2014). Of relevance, hnRNP A1 is also critically involved in the exon skipping of survival motor neuron 2 (*SMN2*; Kashima et al., 2007), involved in spinal muscular atrophy (SMA), another form of motor neuron disease and hnRNP A2/B1 has been implicated in Alzheimer’s disease (Mizukami et al., 2005). hnRNP A2/B1 and hnRNP A1, as well as TDP-43, have intrinsically disordered, aggregation-prone domains at their C-terminal end (Kim et al., 2013) supporting the contention that these proteins are prone to fibrillization and misfolding. Further, TDP-43 depletion leads to upregulation of specific isoforms of hnRNP A1, one of which has been reported to be highly prone to aggregation (Deshaies et al., 2018). Another RNA-binding protein involved in *MBP* trafficking is QKI; QKI mutant mice actually have impaired nuclear export of *MBP* mRNA resulting in impaired myelination in these mice (Larocque et al., 2002). QKI was characterized to interact with hnRNP F and H during alternative splicing in myelinating glia (Mandler et al., 2014). Further, post-mortem samples from ALS patients harboring *C9orf72* mutations demonstrated that RNA foci sequester hnRNP H/F (Cooper-Knock et al., 2014); hnRNP H/F is necessary for PLP/DM20 alternative splicing (Wang et al., 2007, 2008), a process critical for normal myelination. Of relevance, in human post-mortem tissue, it has been shown that ALS patients have a significant reduction in MBP in both the motor cortex and ventral spinal cord (Kang et al., 2013); however, it remains unclear as to whether the reduced myelin is a downstream effect of axonal loss or whether it is an intrinsic deficit in oligodendrocytes.

Oligodendrocytes have a very low annual turnover of just 1 in 300 oligodendrocytes in humans, making them a very stable cell type in the CNS (Yeung et al., 2014). The production of myelin, on the other hand, is a more dynamic process (Yeung et al., 2014) with one cell possessing the ability to produce three times its own weight in myelin per day, eventually supporting 100 times its own cell weight in myelin (McLaurin and Yong, 1995; Connor and Menzies, 1996; Ludwin, 1997). Thus, given that an oligodendrocyte also has to metabolically support neurons in addition to their own metabolically demanding myelination requirements, this makes them vulnerable to processes that may interfere with myelin modulation. Given that many of the RNA-binding proteins that are critical for myelin-related mRNA trafficking in oligodendrocytes have been implicated in ALS pathology, this poses a risk to cellular function and their ability to support neurons.

## Conclusion

It is well established that the hallmark pathological signature of ALS is misfolding and accumulation of aggregation-prone proteins, the most common of these proteinopathies being TDP-43. To date, very little attention has been given to the role of glia, specifically astrocytes and oligodendrocytes, in the pathogenesis of ALS as a result of intrinsic cellular deficits arising from protein aggregation. It is known in neurons that pathological protein aggregation, as well as the formation of RNA foci in *C9orf72* cases, can have a toxic gain-of-function effect by sequestering other mRNAs and proteins, compromising normal cellular function. We suggest this process is also occurring in glia and is complicated by the fact that many critical mRNAs required for normal astrocytic and oligodendroglial function are locally translated. Given the association of many RNA-binding proteins with the typical protein aggregations found in ALS cases, and the known associations of these RNA-binding proteins with key mRNAs in astrocytes and oligodendrocytes, we suggest that this is a potential pathogenic pathway of disease contributing to cellular dysfunction in patients with ALS.

## Author Contributions

SB, JG, SC and BT contributed to the preparation, writing and editing of this review article.

## Conflict of Interest Statement

The authors declare that the research was conducted in the absence of any commercial or financial relationships that could be construed as a potential conflict of interest.
